# Supplementary material for: Transcriptomic and Proteomic Analysis of CRISPR/Cas9-Mediated ARC-Knockout HEK293 Cells
Source: Int J Mol Sci. 2022 Apr 19;23(9):4498. doi: 10.3390/ijms23094498 (PMC9101110; doi:10.3390/ijms23094498)
Supplement: Supplementary file 1 [file ijms-23-04498-s001.zip › Supplementary Figure S1.pdf]

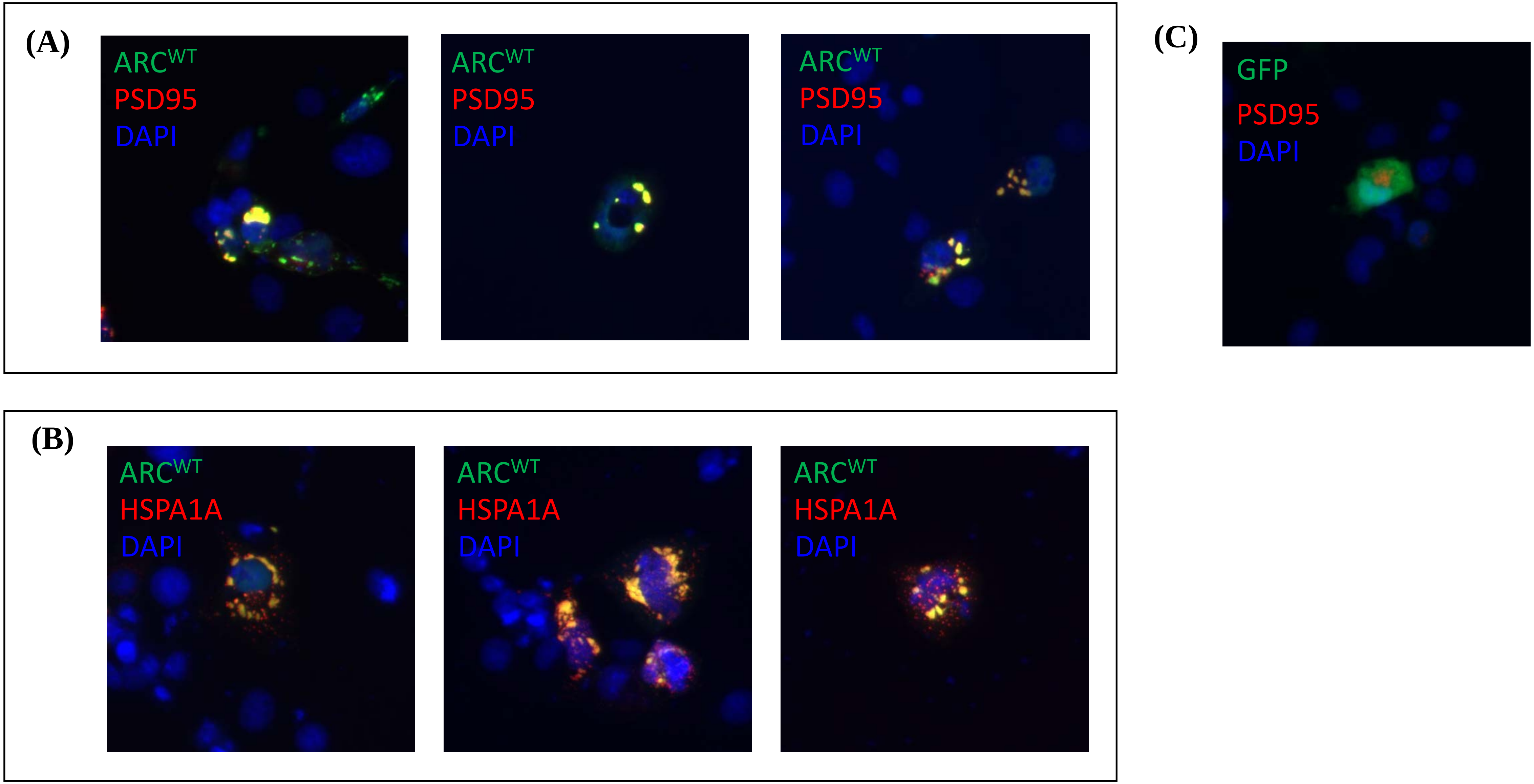

Supplementary Figure S1. ARC^{WT} (green) colocalized with (A) PSD95 (red) and (B) HSPA1A (red) in transfected COS-1 cells. (C) GFP (green) did not colocalize with PSD95 (red). DAPI (blue), 4',6-diamidino-2-phenylindole.
