# Supplementary material for: Transcriptomic and Proteomic Analysis of CRISPR/Cas9-Mediated ARC-Knockout HEK293 Cells
Source: Int J Mol Sci. 2022 Apr 19;23(9):4498. doi: 10.3390/ijms23094498 (PMC9101110; doi:10.3390/ijms23094498)
Supplement: Supplementary file 1 [file ijms-23-04498-s001.zip › Supplementary Table S1.pdf]

Supplementary Table S1. The off-target sites for *ARC* gRNA.

Location	Number of mismatches	Sequence (including mismatches)
chr19:50469223	3	CCC**t**AGCACCT**tt**GCACAGATGG
chr2:200696040	3	CCAGAGCA**g**CT**c**CGCACAGAT**t**G
chr21:31356832	3	CCATCTG**g**GCGCAG**tg**GCTCAGG
chr4:6172657	3	CC**t**TCTGT**c**C**c**CAGGTGCTCAGG
chr9:93267363	3	CCTG**g**GCACCTG**t**GC**c**CAGATGG
chrX:68878063	3	CCGG**t**GCACC**a**G**g**GCACAGATGG

Small bold letters mean mismatch nucleotide.
