# Supplementary material for: Transcriptomic and Proteomic Analysis of CRISPR/Cas9-Mediated ARC-Knockout HEK293 Cells
Source: Int J Mol Sci. 2022 Apr 19;23(9):4498. doi: 10.3390/ijms23094498 (PMC9101110; doi:10.3390/ijms23094498)
Supplement: Supplementary file 1 [file ijms-23-04498-s001.zip › Supplementary Table S2.pdf]

Supplementary Table S2. RNA read summary of *ARC*-KO HEK293 cells (KO) and wild type HEK293 cells (WT) libraries.

**Sample**	**Total Reads**	**Unmapped**	**Total Mapped**	**Multiple Mapped**	**Uniquely Mapped**	**Splice reads （UM）**	**Non-splice reads （UM）**
ARC-WT_1	43171932 (100 %)	722881 (1.67 %)	42449051 (98.33 %)	2677367 (6.2 %)	39771684 (92.12 %)	24409895 (56.54 %)	15361789 (35.58 %)
ARC-WT_2	40147588 (100 %)	681602 (1.7 %)	39465986 (98.3 %)	2218278 (5.53 %)	37247708 (92.78 %)	22453145 (55.93 %)	14794563 (36.85 %)
ARC-WT_3	40411842 (100 %)	806274 (2 %)	39605568 (98 %)	1987502 (4.92 %)	37618066 (93.09 %)	22645360 (56.04 %)	14972706 (37.05 %)
ARC-KO_1	42238834 (100 %)	639661 (1.51 %)	41599173 (98.49 %)	2122187 (5.02 %)	39476986 (93.46 %)	23883427 (56.54 %)	15593559 (36.92 %)
ARC-KO _2	46470614 (100 %)	676768 (1.46 %)	45793846 (98.54 %)	2283717 (4.91 %)	43510129 (93.63 %)	26935132 (57.96 %)	16574997 (35.67 %)
ARC-KO_3	44057278 (100 %)	636571 (1.44 %)	43420707 (98.56 %)	1937483 (4.4 %)	41483224 (94.16 %)	25016603 (56.78 %)	16466621 (37.38 %)
