# Supplementary material for: Transcriptomic and Proteomic Analysis of CRISPR/Cas9-Mediated ARC-Knockout HEK293 Cells
Source: Int J Mol Sci. 2022 Apr 19;23(9):4498. doi: 10.3390/ijms23094498 (PMC9101110; doi:10.3390/ijms23094498)
Supplement: Supplementary file 1 [file ijms-23-04498-s001.zip › Supplementary Table S4.pdf]

Supplementary Table S4. The number of identifications in each sample.

Sample	Protein ID	Peptide ID	PSM	Search input
ARC-WT_1	813	6025	12481	20181
ARC-WT_2	828	6056	12655	20260
ARC-WT_3	836	6017	12549	20177
ARC-KO_1	850	6180	12621	20053
ARC-KO_2	820	6154	12634	20034
ARC-KO_3	832	6101	12574	20022
